# Use of the Mechanical Leech for Successful Zone I Replantation

**DOI:** 10.1155/2014/105234

**Published:** 2014-03-23

**Authors:** Sang Wha Kim, Hyun Ho Han, Sung-No Jung

**Affiliations:** Department of Plastic and Reconstructive Surgery, Uijeongbu St. Mary's Hospital, College of Medicine, The Catholic University of Korea, 271 Chenbo-ro, Uijeongbu-si, Gyeonggi-do 480-717, Republic of Korea

## Abstract

Replantation of zone I finger injuries remains a challenge, particularly if the fingertip was previously scarred or atrophied, which makes it difficult to secure a suitable vein at the amputation site. In cases of artery-only anastomosis, we propose using a mechanical leech technique to maintain sufficient venous outflow until the internal circulation regenerates. We applied this procedure to eight patients who had zone 1 amputations without veins that were suitable for anastomosis. Emergent surgery was performed and an artery-only anastomosis was created. As there were no veins available, we cut a branch of the central artery and anastomosed it with a 24-gauge angioneedle, which served as a conduit for venous drainage. The overall survival rate for zone I replantation using mechanical leech was 87.5% and the average time to maintain the mechanical leech was 5 days. The mechanical leech technique may serve as an alternative option for the management of venous congestion when no viable veins are available.

## 1. Introduction

Although microsurgical techniques have advanced and been refined since the first finger replantation 40 years ago [1], replantation of zone I finger injury beyond the distal interphalangeal joint remains a challenge. As the amputation site becomes more distal, the diameter of the vessels decreases making anastomosis more technically difficult. Especially in previously injured fingers, often only a solitary artery is available for arterial inflow without a suitable vein for sufficient venous outflow. As a result, venous congestion is often responsible for failure of replantation.

Previous reports have described methods to accommodate venous drainage without creating an anastomosis [2–7]. We presently introduce the mechanical leech technique for relief of venous congestion in replantation, which has been previously used to alleviate venous congestion of flaps [8–10]. This technique is used to maintain sufficient venous outflow until internal circulation regenerates after replantation. Presently, we describe the mechanical leech technique and report successful replantation using this method.

## 2. Patients and Methods

From January 2011 to December 2012, 22 zone I replantations were performed. Among them, 14 patients who had venous anastomosis were excluded and eight patients were included in this study. The eight patients comprised six males and two females (25–56 years of age, mean age, 40.9 years). All the injuries were complete amputations involving one thumb, one index finger, three third fingers, two ring fingers, and one little finger. The average followup was 16.3 months (range, 10–26 months). Detailed patient characteristics are presented in Table 1.

### 2.1. Surgical Procedure

**Figure 1:**
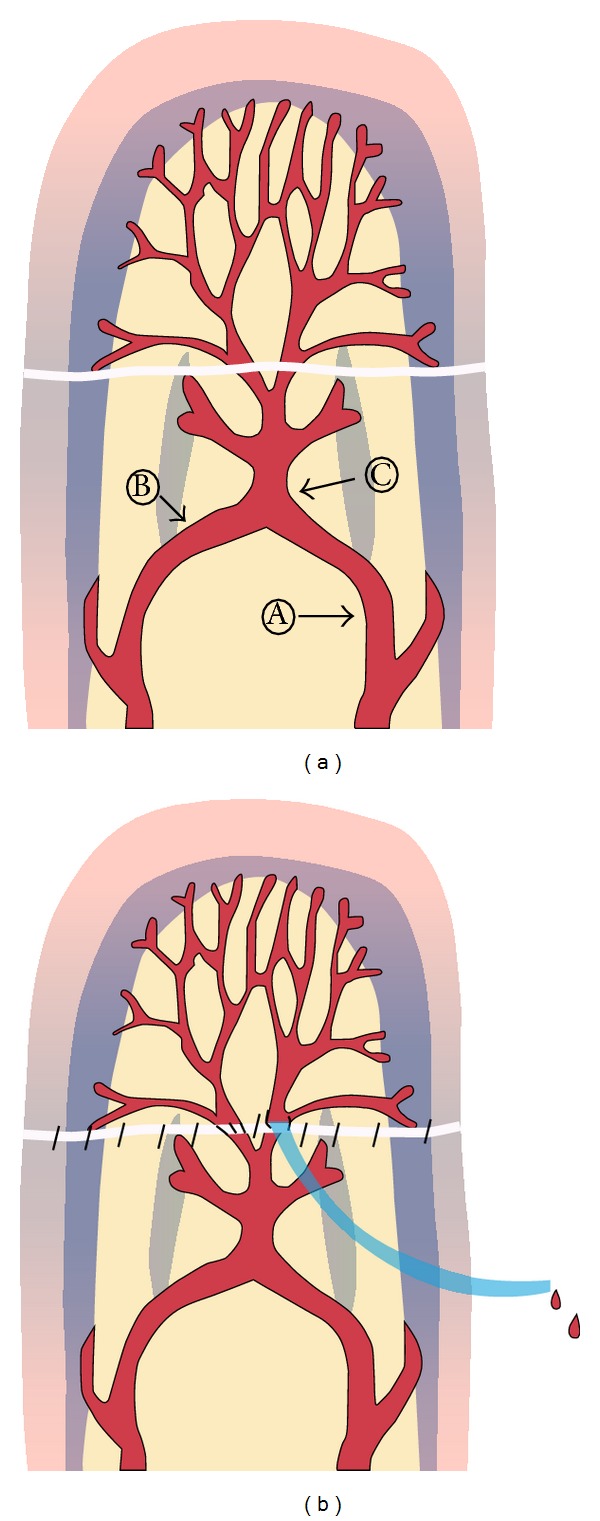
Details of the mechanical leech. (a) Schematic of the mechanical leech. A: Proper digital artery, B: distal transverse arch, and C: central artery. (b) After the arterial anastomosis, the remaining branch of the central artery, which drains after the artery inflow, was easily identified. The angioneedle was anastomosed to the branch and functioned as a conduit for venous drainage.

Under general anesthesia, careful debridement of the amputated digit was performed and bony fragments were fixated with a K-wire. Under microscopic guidance, the neurovascular bundle was dissected to locate an available vessel. The artery was anastomosed using 10-0 nylon. Venous outflow was subsequently identified, and in cases where no vessels were available, dissection was performed more distally along the digital artery to identify the central artery. A branch of the central artery was identified to be utilized as venous drainage. The angioneedle was chosen according to the size of the vessel. The angioneedle was inserted through the skin of the distal stump of the finger and anastomosed to the branch using two stitches (10-0 nylon) positioned, 180° apart (Figure 1). The skin was closed using 5-0 nylon. After surgery, the patient was monitored and the external opening of the catheter was covered with heparin gauze and flushed with heparinized saline (500 IU/cc) every hour to prevent clotting of the catheter. Additionally, heparin was continually administered intravenously (5000 IU every 24 hour) for 3 days. The catheter was removed 4–7 days after the operation once vascular stability had been achieved.

## 3. Results

The overall survival rate for zone I replantation using the mechanical leech was 87.5%. Seven of eight cases survived completely. One case demonstrated venous congestion, even though the mechanical leech was maintained for 7 days postoperatively. The average time to maintain the mechanical leech before reestablishment of venous drainage was 5 days.

### 3.1. Case 1

**Figure 2:**
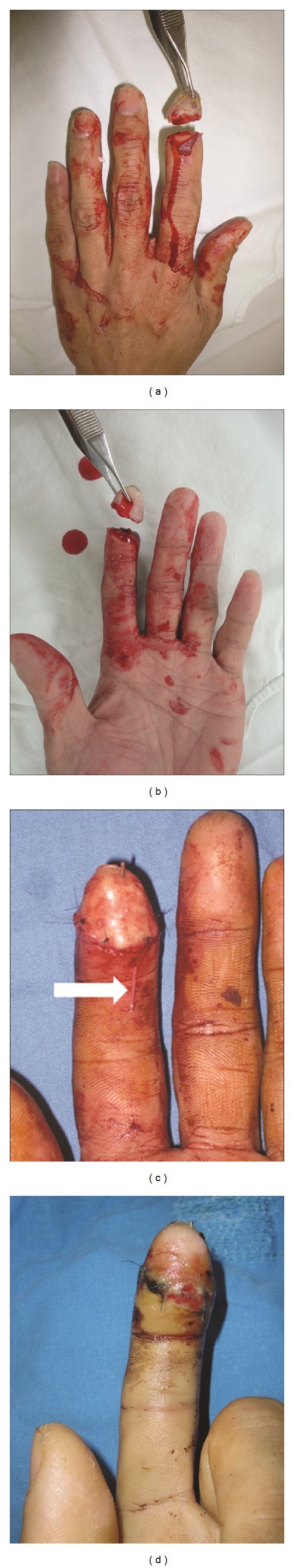
Details of Case 1. (a) and (b) A 46-year-old man amputated his left second finger just distal of distal interphalangeal joint. The patient had history of blunt trauma with a hammer to the same finger leaving the amputation site scarred and atrophied. (c) Immediate postoperative view. The angioneedle is located proximal to the amputated portion (arrow), which was anastomosed to the branch of central artery and functioned as a conduit for venous drainage. (d) View on postoperative day 8. The digit was replanted successfully.

A 46-year-old male presented 30 minutes after injuring his left second finger in a candy making machine. The patient had sustained blunt trauma by hammer to the same finger one month previously but had not received any treatment. On physical examination, a clean amputation just distal to the distal phalangeal joint of the second finger in zone I was observed. The finger was scarred and atrophied due to the previous trauma (Figure 2(a)). Emergent surgery was performed using the mechanical leech procedure with a 24-gauge angioneedle since no viable veins were available for anastomosis (Figure 2(b)). After the surgery, the catheter was flushed with heparinized saline every hour, and heparin was continually administered intravenously over 3 days. The patient was hemodynamically stable and did not require a blood transfusion. The catheter was removed on postoperative day 4. The amputated digit was replanted successfully without arterial insufficiency, venous congestion, or infection (Figure 2(c)). The patient was satisfied with the result and returned to work 2 months after the operation.

### 3.2. Case 2

**Figure 3:**
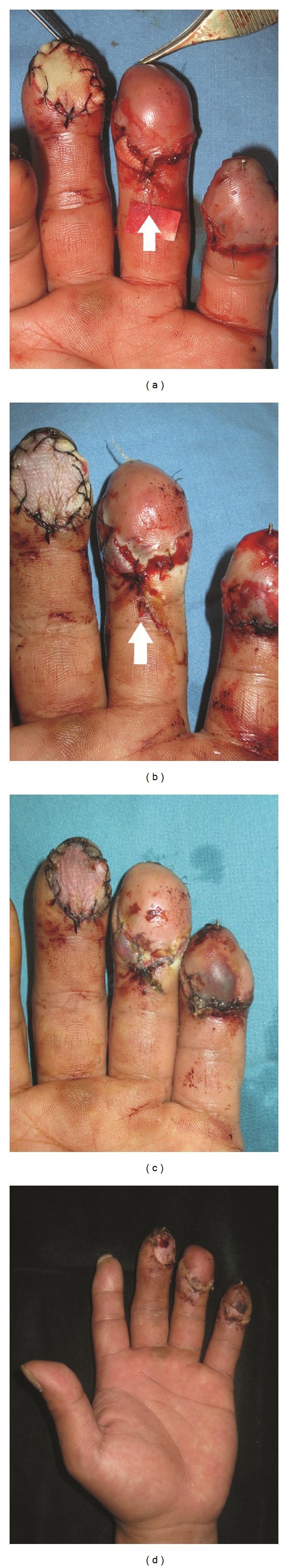
(a) Details of Case 2. (a) Immediate postoperative view. A full-thickness skin graft was done on the third finger, and the fifth finger was replanted via anastomosis of one artery and one vein. The mechanical leech procedure using an angioneedle (arrow) was performed on the fourth finger. (b) View on postoperative day 4. The angioneedle is located proximal to the amputated portion (arrow). (c) The patient was discharged on postoperative day 7. The angioneedle was removed on postoperative day 5, and all digits were reconstructed successfully. (d) 3 weeks after the operation. The patient was encouraged to exercise and had gone back to his normal daily routine.

A 42-year-old male employee at a textile factory suffered a crushing injury of the third, fourth, and fifth fingers. The patient had a history of multiple fingers injuries sustained at work. On physical examination, a volar oblique amputation in zone I of the fourth and fifth digits and a skin defect of the third digit were observed. Emergent surgery was performed. The skin defect on the third finger was repaired with a full-thickness skin graft from the ankle. Replantation was performed on the fifth finger with anastomosis of one artery and one vein. There were no viable veins in the fourth finger, so the mechanical leech procedure was performed (Figure 3(a)). After the surgery, the catheter was flushed with heparinized saline once per hour, and heparin was continually administered intravenously over 3 days. The catheter was maintained for 4 days and removed on postoperative day 5 (Figure 3(b)). All of the digits were replanted successfully (Figure 3(c)).

## 4. Discussion

Successful replantation of a digit depends on the establishment and maintenance of blood flow across the arterial anastomosis, fingertip, and venous outflow. Although an amputated fingertip seems possible to be replaced by a composite graft, there is limited success in children, not in adults, and the results are unpredictable. Restoration of circulation by microsurgical anastomosis is the only method that ensures success [8].

Success rates for replantation distal to the distal phalangeal joint are as high as 70–90% when both the artery and vein are repaired [2]. However, it is often difficult to secure a suitable vein in zone 1 amputations, especially if the finger has previously been injured.

When adequate venous anastomosis cannot be performed, various methods have been described to maintain the flow until internal circulation is achieved. One alternative method to reestablish internal venous outflow is an arteriovenous shunt [4]. This technique is useful for venous drainage if a second artery can be found at the amputation site and an appropriate vein can be found at the distal stump. In these cases, vein grafting is typically needed to connect the secondary artery and the vein. Another technique for establishing venous drainage is the use of a cutaneous-venous fistula [3], which is constructed from a vein graft 3 cm in length and 2-3 mm in diameter. The vein graft is placed between an open punch wound on the volar side of the replanted fingertip and a vein in the dorsum of the finger. There is little data, however, regarding the extent of venous drainage or success rates of cutaneous-venous fistulas. Finally, delayed venous drainage is a technique in which the artery is anastomosed first, and the vein is subsequently anastomosed 8–12 hours later [5]. Although this is a simple technique with a proven high success rate, the need for a second operation is a major drawback.

If internal venous drainage cannot be established, there are several methods that utilize external bleeding to reduce venous congestion until internal circulation is established. Bleeding techniques have been described that use a fish mouth incision or skin defect in the distal fingertip in conjunction with topical heparin. This requires frequent dressing changes and monitoring due to the potential for uncontrolled bleeding or clotting. Another incision is often needed if blood clots develop. Secondary injury due to the incision or skin defect is inevitable, which may result in sensory loss, scarring, or atrophy of the replanted fingertip when the replantation itself is considered to be successful. Medical leeches are also available and commonly used to establish external venous drainage through the skin. However, venous drainage using medical leeches also has several drawbacks, such as infection with organisms such as* Aeromonas, Pseudomonas*, and* Staphylococci* in 7 to 20% of cases [9]. Positioning of the leech may be difficult, especially in small areas such as the fingertip. It is difficult to control the volume of blood loss when using medical leeches, at times leading to the need for blood transfusion to salvage a small portion of the digit. Moreover, this method tends to be less appealing to the patient and family members. Recently, a subdermal pocket procedure for difficult fingertip replantation has been reported [6]. In this method, the deepithelialized pulp surface of the replanted fingertip is inset into an abdominal pocket using the rich venous channels of the subdermal plexus. This technique requires physical restriction period for 1-2 weeks and a permanent scar remains at the donor site in the abdomen.

The mechanical leech technique has been introduced to salvage free flaps [10] or local flaps [9, 11] with venous congestion. Venous congestion was successfully relieved by placement of the mechanical leech consisting of a silastic rubber catheter, 3-lumen central venous catheter, and simple 16- to 18-gauge venous catheter connected to the veins of the flap. This catheter drains the excess blood in conjunction with systemic anticoagulation therapy.

In the finger, there are three major palmar arterial arches: the proximal arch and middle arch are located at the proximal and distal cruciate ligaments and the distal arch lies just distal to the insertion of the profundus tendon. The central artery originates from the distal arch and travels longitudinally to supply the pulp. The diameter of the arch is reported to be 0.85 mm and the central artery to be approximately 0.58 mm [12, 13]. In case of zone I amputation, we could find central artery at the pulp under microscopic examination and meticulous dissection along the central artery could identify the undamaged lesion and could trace the branches from the central artery [7]. For the mechanical leech technique, the angioneedle is anastomosed to the branch of the central artery, functioning as a conduit for venous drainage. Although presently the catheter was flushed with heparinized saline every hour, systemic administration of heparin was necessary to prevent blood clots. It took 3-4 days to achieve vascular stability, which was confirmed by color and capillary refill of the nail bed. Systemic administration of heparin did not affect the hemodynamic status of the patient and transfusion was not required. The catheter was removed by gentle pressure, without any additional bleeding.

There are several advantages to using the mechanical leech. Perhaps most importantly, this method can be used for replanted fingers without suitable vessels for venous anastomosis. Since various types and sizes of catheters are available, this method can be tailored to the available vessels such as the cruciate anastomosis. While traditional bleeding techniques or the medical leech carries a significant risk of infection, the mechanical leech utilizes a cleaner surgical procedure. Moreover, it does not require additional dressing, subsequent surgical procedures, or additional wounds. Heparin flushes into the lumen of the catheter help to prevent clotting with minimal bleeding. Profuse bleeding, such as that seen in bleeding techniques that use a fish mouth incision or skin defect, is unnecessary.

## 5. Conclusion

We introduce a simple mechanical leech technique using an angioneedle, which may provide another option for the management of venous congestion in zone I replantation when no viable vessels are available for anastomosis.

## Figures and Tables

**Table 1:** Detailed patient characteristics.

	Age	Sex	Diagnosis	Mechanical leech period (days)	Replantation success	Complications	Followup (months)
1	46	M	Amputation, 2nd finger, Lt.	4	Total survival	None	26
2	39	M	Amputation, thumb, Rt.	7	Total survival	None	25
3	45	F	Amputation, 4th finger, Rt.	4	Total survival	None	16
4	56	F	Amputation, 5th finger, Lt.	7	Fail	Venous congestion	16
5	42	M	Amputation, 3rd finger, Rt.	5	Total survival	None	13
6	25	M	Amputation, 3rd finger, Lt.	4	Total survival	DIP joint stiffness	12
7	32	M	Amputation, 3nd finger, Rt.	5	Total survival	None	12
8	42	M	Amputation, 4th finger, Lt.	4	Total survival	None	10
